# The contribution of quality management practices to student performance: Mediated by school culture

**DOI:** 10.1016/j.heliyon.2024.e34892

**Published:** 2024-07-19

**Authors:** Khalida Parveen, Tran Quang Bao Phuc, Abdulelah A. Alghamdi, Tribhuwan Kumar, Sarfraz Aslam, Muhammad Shafiq, Atif Saleem

**Affiliations:** a College of Teacher Education, Qujing Normal University, Yunnan, China; b Faculty of Foreign Languages, Ho Chi Minh City University of Foreign Languages and Information Technology, Vietnam; c Department of Educational Policies, Faculty of Education, Umm al-Qura University, Makkah, Saudi Arabia; d Department of English Language and Literature, College of Science and Humanities at Sulail, Prince Sattam Bin Abdulaziz University, Al Kharj, Saudi Arabia; e Faculty of Education and Humanities, UNITAR International University, Malaysia; f School of Information Engineering, Qujing Normal University, Yunnan, China; g School of Education, University of Limerick, Ireland

**Keywords:** Quality of education, Quality management practices, School culture, Student performance

## Abstract

School management is responsible and accountable for implementing educational policies into practice effectively and efficiently to provide quality education. Simultaneously, school management can grasp the core features of the whole school process and identify the relationship among three variables: quality management practices, school culture, and student performance. The current study aims to explore the school principals' perception about quality management practices and its relationship with school culture and student performance in the public secondary schools of Punjab province, Pakistan. In order to achieve the objectives of the study, the study adopted an exploratory sequential mix-methods research design. The researcher conducted a systematic literature review of sixty-three previous studies and interviews with eleven school principals for the qualitative data. Based on results obtained from the qualitative phase, a questionnaire was prepared and dispatched to 150 school principals to get quantitative data. Successively 120 valid responses were received. SEM analysis was performed to get quantitative results. The study's preliminary conclusion demonstrated a positive connection between quality management and student performance in public secondary schools, and quality management was also a significant predictor of school culture. Further, school culture served as a complete mediator between quality management and student performance.

## 1 Introduction

### 1.1 QM and education

Quality management (QM) covers all operations and functions aimed at achieving quality, also known as quality assurance by most QM advocates [1]. It is a comprehensive strategy that aims to achieve excellence in all operational terms of the institution through collaboration between every organization's elements and all components [2]. Furthermore, QM enables an institution, organization, or company to accomplish its goals more effectively while maximizing its personnel's capacity for ongoing improvement [3].

The most important ability for any country to be beneficial for achieving significant social and economic change is access to high-quality education at all levels of schooling. Further, the approach to high-quality education is the most important factor in determining young generations' long-term development/competency and a nation's future. To make this vision a reality, it is necessary to talk about what attributes to excellence in educational systems and across all educational levels [4,5].

In fact, good administration, management, and school discipline can guarantee the efficiency and excellence of school premises [6]. Today's social expectations for a principal include doing several tasks to improve the educational environment [4,7]. As per Isaksson [8], administrators make sure that academic and administrative tasks are goal-oriented, that academic staff adheres to stringent guidelines for instructions and curriculum development, and that the institution is efficiently run while also being accountable for all matters of the school. Also, good schools have an effective and enthusiastic administration that is committed to fostering development with a good philosophy, clear vision, teamwork, creativity and innovation, and attention to ongoing improvement [[9], [10], [11]].

The primary goal of educators who prepare the next generation for success, according to Köksal, Batmaz [12], is to create a quality environment; thus, school administrators and teachers should utilize quality practices in their work with students. However, it can be achievable if the school administration successfully tries to cope with and support the chores involved in school management and leadership [13]. To maximize organizational effectiveness and QM, the primary approaches comprise monitoring the educational program, satisfying and cultivating staff, restoring the school organization, improving the school climate, etc. Introducing QM, or components of continuous improvement, is one of the primary ways to assist them in raising their standards and maintaining the value of their services [14].

An extensive review of related literature indicates that applying QM concepts is one of the initiatives to assess and improve educational quality [[15], [16], [17], [18], [19]]. The implementation of QM principles has also provided a competitive advantage to local educational institutions [20,21]. Accordingly, essential components supported by QM are a strong sense of school vision, boosting daily management, producing an improved staff, focusing on student-driven values, and developing achievable goals [16].

### 1.2 Pakistan education challenges

School education of the highest caliber was designated a fundamental right of every person in the Human Rights Universal Declaration of 1948 [22]. Additionally, the Pakistan National Educational Policy strives to increase retrieve to education by pondering and increasing retention on lower dropout rates, enhancing secondary education's quality through increased enrollment, and enrolling students in order to meet national educational goals [21].

The entire educational system in Pakistan is inevitably impacted by many challenges already associated with the whole system, particularly those related to secondary school management, and have an impact on student learning. Although the country has introduced QM standards in local educational institutions, school quality and student performance are not as satisfactory as the government hoped. Accordingly, there is a growing competition between private and public schools in terms of educational quality. Local public schools must develop novel strategies to overcome the difficulties that the new millennium has brought in order to compete with the private sector.

Currently, in Punjab, the most populated and literate province, school management has to deal with many problems in terms of educational quality and equity. The big problem is the move from public school students to private schools. The dropout rate in public schools is continuously growing due to the lower quality of public schools. Although there are many outstanding aspects of government schools, many people still believe that the private sector provides a better education, probably because it is better funded. Therefore, intellectual students prefer to get admission to private schools and leave public schools [8].

There are various reasons for the deterioration of educational quality in public schools. These reasons include the absence of accountability and transparency mechanisms, teachers' absenteeism, corporal punishment, political influence in the transfers and postings of teachers, and all of the above deficiencies in administration and management. Further, perhaps the curricula that are mandated for schools are also problematic since the curricula are not created by experts in the educational system and frequently do not correspond to contemporary trends. Teachers are given difficult goals to complete and are forced to teach using formal curricula with insufficient resources. Unfortunately, not all amenities are offered in public schools. Educational quality at the basic and secondary levels received little attention in the past since multidisciplinary courses were entrusted to a single teacher with an irrelevant educational background with the idea that they would be taught successfully and efficiently. As a result, public schools now face a significant quality problem and the level of quality is insufficient [23]. Parents are willing to pay to enroll their kids in a private school for excellence.

Likewise, a key motivation for our study is the huge difference between the student outcomes of public and private secondary schools [24]. The Board of Intermediate and Secondary Education (BISE) is liable for handling annual exams of secondary schools for grades 9th, 10th, 11th, and 12th. The results provided by (BISE) during the past five years show that private schools have higher grades than public ones. The most recent yearly results of the Punjab Boards attest that private schools have taken all top positions [20].

Though the government of Punjab province of Pakistan has mandated free and universal secondary education, it won't be simple to establish universal education with any degree of equity [25]. There are variations between urban and rural communities, and the significance of education is not always recognized. Parental poverty and illiteracy are problematic in rural communities. As a result, girls have a low literacy rate, particularly those of poverty-stricken communities. Accordingly, rural girls enroll at a rate that is 45 % lower than urban girls, whereas the differential for boys is only 10 %.

At provincial level, in 2010, a whole-system reform was designed and launched in the Punjab called the Punjab Schools Reform Roadmap (PSRR). The focus of the reform was to enhance the quality of public schools. This initiative was launched to improve the quality of education in the province while also analyzing the outcomes and sustainability of the reform ten years from its inception. This reform, which included three dimensions: quality management, teacher capacity, and monitoring and information systems, directly responded to education challenges in the province at the time. As QM is the first dimension of the PSRR, the researcher was motivated to conduct the study on QM practices in the context of educational reform in Pakistan.

This current research paper's goal is to investigate the growing interest in QM in education and its effect on student performance with the mediating effect of school culture in the context of Punjab province of Pakistan's secondary education. The study's motivation comes from the Punjab province's secondary schools' attempt to address the current challenges in terms of educational quality and equity, and the local education system's attention/pursuit to Sustainable Development Goal No. 4 (SDG 4–2030).

### 1.3 Research question & hypotheses

The following research question and hypotheses have been formulated to get the objectives of the study:

RQ. What is the perception of the school principals about the relationship among quality management practices, school culture, and student performance?

A literature review was conducted to generate hypotheses relating Quality Management to Student Performance and School Culture. SEM (Structural Equation Modeling) requires developing hypotheses based on literature reviews. Having thoroughly examined previous research on the relationships among these essential elements, we formulated our hypotheses.

H1 Quality management practices and student performance show a positive and significant relationship.

H2 Quality management and school culture depict a significant and positive relationship.

H3 School culture and student performance indicate a significant and positive relationship.

H4 School culture has a positive mediating effect on the relationship between quality management and student performance.

## 2 Literature review

### 2.1 Quality management and education

Quality gurus like Scherkenbach [26] and Shewhart and Deming [27] have transformed their beliefs into standardized 'Best Practices' or 'Quality Models' which are a great source of insight and direction for managers all around the world [28]. According to Nakhai and Neves [29], the primary characteristics of the QM models are an emphasis on continuous improvement, customer happiness, teamwork, employee interactions, and customer involvement. The six core principles on which QM is built are focusing on customers, continual improvement, focusing on processes, making decisions based on facts, everyone's dedication, and dedicated leadership [30].

**Fig. 1:**
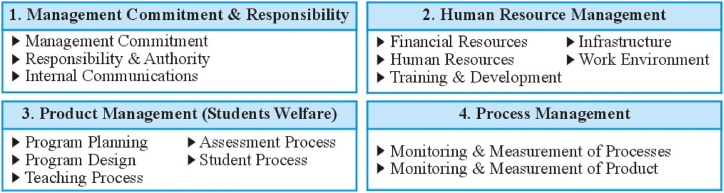
Domains of ISO 9000.

There are different kinds of these models and frameworks established worldwide by organizations such as i) The Malcolm Baldrige National Quality Award (MBNQA: 2011–2012), ii) the Prime Minister Quality Award (PMQA), iii) The European Foundation for Quality Management (EFQM), and iv) the International Standards Organization (ISO). The current study adopted a theoretical framework based on the [27] Model for Quality Management in education (1986) Dr. Joseph M. Juran's Quality theory (1904), and the International Organization for Standardization (ISO) 9001 Quality Management System. As Shewhart and Deming's and Dr. Juran's theory of quality deliver the foundation (theoretical background), whereas the ISO 9001 Quality Management Model is the operational aspect (practical background) of the quality management framework for educational institutions. Many previous studies have used the same ISO 9001, QM dimensions (Fig. 1) [[31], [32], [33]].

Manufacturing businesses have found QM quite useful, and service organizations are now starting to adopt it as well. There are many QM Models available, but there are few clear instructions on what to do and what is necessary to adopt QM successfully in the field of education. A thorough understanding of these models may be useful for implementing QM in schools. Since the ideas behind QM and quality control have their roots in management and industrial sciences, and the models used for quality control in education are mainly grounded on the same mindset. Then, these models were modified and used in educational contexts.

Though QM has successfully raised productivity and provided higher-quality services while boosting employee and student morale, it was stated in previous literature that the significant obstacle to implementing QM philosophy in schools is the misapprehension of its philosophy. Due to several factors, such as incompetence on the part of the school administration and poor record keeping, it is not easy to assess the quality of academic operations. The management of schools and colleges chooses only a small number of key components of QM, typically based on personal preferences. As a result, it is necessary to adopt several models in order to assess educational quality.

Globally, several schools have used QM differently [34]. There are a few frameworks that have been expressly designed for education. According to the available literature [[35], [36], [37]], there is growing interest in the application of QM in the education sector for a variety of reasons, some of which are: i) industry pressure to raise academic standards in light of technological advancements; ii) government-funded programs for teaching & research in the quality area; iii) to get a good reputation as funding for research and teaching is given only to the more reputable institutions; iv) increased competition between private and public academic institutions.

Education is the foremost component for boosting the nation's socioeconomic improvement and development and equipping future generations with information and skills. The nations can improve people's social, economic, emotional, mental, and psychological development in proper manners by delivering high-quality education. Educational quality is defined as the adherence to educational metrics fixed by the institution, like; enrollment rate, graduation rate, mode and manner of instruction, resource accessibility, and infrastructure. It should be the duty of any society in the world to focus on maintaining and raising education standards.

Furthermore, in today's world, quality education is becoming increasingly important as it integrates with industry and changes due to technological advancement. Social media and teleconferencing have transformed education outside the traditional classroom by giving students and teachers access to virtual classrooms and interactive groups. During the COVID-19 epidemic, only educational institutions with pre-existing IT-based schooling systems could maintain the abrupt transition in educational delivery modalities from on-campus to online [[38], [39], [40]]. Teachers have been strained to improve their teaching strategies and raise the standard of education; as a result, students have access to information at their fingertips.

For the quality-conscious age we live in, education serves a vital purpose. Quality education makes learning enjoyable [12]. Shewhart and Deming [27], Harris [41], and Middlewood [42] examined the application of quality criteria in education and concluded that they are crucial to boosting the field's standards. Accordingly, the theory of QM in education emphasizes improvement, enhancement, and transformation for all student services [16]. QM aims to provide students with high-quality instruction inside the institution [43]. It could help a school improve the services it offers to its students, employers, and other stakeholders [44]. A school's efforts to better serve its main clients, students, employers, and other stakeholders could be aided by QM.

### 2.2 Quality management and student performance

Academic performance gauges a student's receptive and indicative skills in relation to what they are learning as a result of their education or training [40]. In other words, a student's academic success involves achieving the aims and goals set forth in the program or course that they are enrolled in. Likewise, examination and their outcomes are a sound determination of quality because they show how well students' knowledge and skill levels align with well-established standards. They are demonstrated by grades that come from assessments that involve passing or failing particular exams, subjects, or courses [45].

An extensive review of literature [46,47] in Nigeria [48], in Bangladesh [49], in Indonesia [50], in Kenya, and [51] in the US indicates that QM can have a significant impact on the performance of school students. On the whole, QM can play a crucial part in improving student performance by promoting a positive learning environment, enhancing the teaching-learning process effectiveness, and facilitating ongoing assessment [[52], [53], [54]].

By implementing effective QM practices, schools could provide an enriched environment to raise student success. QM aims to provide students with high-quality instruction inside the institution [43]. The quality of the process is the reflection of the delivery process, which largely measures what happens in the common classroom and throughout the school as a whole. QM could lead to better attention to student success and developing strategies to support student growth. QM could help ensure that teachers deliver lessons effectively and students actively engage with the material.

Also, QM could encourage teachers to find new and innovative ways to keep students engaged in the process of learning. This could lead to greater student motivation and a more enjoyable learning experience. Moreover, QM could help to make sure that assessments and evaluations are reliable, accurate, and consistent. This could provide valuable information to teachers and students on areas where they need to improve, leading to better performance over time. Overall, QM could lead to better student outcomes and more efficient use of resources.

### 2.3 Quality management, school culture, and student performance

Generally, school culture refers to the relationships, attitudes, rules and regulations that reflect the schools' operations. Barth [55] perceives school culture 'a complex pattern of norms, attitudes, beliefs, behaviours, values, ceremonies, traditions, and myths that are deeply ingrained in the very core of the organization'. It also includes many other concrete concerns, including the emotional and physical safety of students and regulations of all public places, i.e., classrooms, libraries, laboratories, and playgrounds.

The thorough examination/review of theoretical models of culture tailored for schools provides a set of attributes of vision and goals, change and innovation, decision-making, modes of communication, teamwork and collaboration, responsibility and commitment/dedication, etc. School culture, as a system of agreed values and norms, gives covenant values of broad criteria and guiding principles for school members to follow in the judgement of the (un)desirability of certain kinds of organizational behaviors, incidents/circumstances, and outcomes. School culture governs how school members make decisions, perceive and manage the organizational environment, handle information, and behave/act in a synchronized manner. Individual commitment and performance may be influenced by school culture, which establishes the norms and practices/ideals for a meaningful workplace environment. A good and enriched culture provides professional satisfaction, efficacy, and morale.

School culture would influence both organizational and personnel performance. Accordingly, the approaches to QM necessitate a school culture built on organizational leaders' and individuals' support, collaboration, and determination to fulfill quality commitments.

The school culture serves as the most important educational foundation that drives student academic success [56]. Review of existing literature reveals that there exists a profound influence of cultural attributes on school outcomes and student performance or a certain correlation between the variables [[57], [58], [59], [60], [61]]. Prior studies by Glušac, Tasic [57] regarding primary schools in Republic of Serbia; Iyabode [58] in the context of public secondary schools in Nigeria; Pervez, Dahar [61] at secondary level in District Rawalpindi, Pakistan, or Melesse and Molla [60] concerning the case of secondary and preparatory schools in Ethiopia are consistent in their findings that school culture has a significant and positive impact on student academic achievement/performance.

According to Khan, Malik [62], most students, teachers, and parents are not satisfied with the quality of instruction at higher education institutes because of inadequate physical and research facilities, poorly stocked libraries and laboratories, and lack of funding. Another study carried out by Jabbar, Hashmi [63] examined the comparison of private and public schools in Pakistan. By selecting a sample of ten universities from the public sector and the same number of institutions from the private sector, he compared the staff quality, quality of students, and quality of infrastructure in public and private schools. According to the survey, private-sector teachers were more competent and self-assured than their counterparts working in public-sector institutions. The private institutions were superior to the public institutions in many other areas, including student quality, infrastructure, and physical facilities like playgrounds, common areas, cafeterias, hostels, and laboratories. However, public and private institutions have been criticized for weak quality management practices.

In a study conducted by Rodríguez-Mantilla, Fernández-Cruz [64], it was determined how two Quality Management Systems (QMS), the EFQM and ISO: 9001 Standards, being implemented simultaneously would affect several predictors of the impact felt by principals. A 91-item questionnaire was administered to a sample of 2869 students from 114 Spanish schools in order to determine how the deployment of QMS was believed to have impacted the classrooms. Hierarchical-linear modeling was used for data analysis. The influence of the results is high in the planning and management system, the external relations, and the learning process. Findings indicated that implementing EFQM had a greater impact than previously thought in schools. The primary goal of this study was to produce empirical data on how people will perceive the effects of the QMS implementation in schools on the climate, learning process, satisfaction, and external relations systems.

Another study by Jabbar, Hashmi [63] explained that quality management is essential for raising student satisfaction. His research examined the ideas behind quality management aspects and how they relate to student satisfaction. In Punjab, Pakistan, secondary school pupils attend public and private educational institutions. A random sample strategy was used to define the population. 727 pupils made up the sample (public 345, private 382). The questionnaire employed a five-point Likert scale to collect data. The study's outcomes demonstrate that the quality of public secondary schools was superior to that of private schools. This paper advises identifying the most important aspects of students' satisfaction with quality control. He continued by stating that quality management is both a mentality and a method for enhancing the educational sector. The training is essential for raising quality management awareness at all levels. Many private schools in Pakistan do not appear to be paying attention to quality management like public sector schools. It is strongly advised that the government create a policy that allows for the inspection of the secondary-level quality management provided to pupils by private educational institutions.

Fundin, Lilja [65] reported that quality management (QM) had demonstrated an excellent capacity for updating and evolution. This study aimed to identify issues determined to be crucial and significant for QM research programs for the next ten years. The study also aimed to launch research for the upcoming Quality 2030 agenda for quality management. This paper is based on considerable data collected throughout a workshop process that was split into two main steps: (1) a collaborative brainstorming workshop with twenty-two academics and practitioners (spring 2019) and (2) an appreciative inquiry summit with twenty researchers and practitioners (autumn 2019). Additionally, five potential study themes for QM were developed as a result of the process: (a) stability in change, (b) systems perspective applied, (c) integrating sustainable development, (d) models for smart self-organizing, and (e) higher purpose as QM booster. The approach also revealed a positive core of QM, which was described as fundamental principles and features of the discipline and practice that must be safeguarded and developed. The outcomes also demonstrated practitioners' support for future QM strategy development. Quality 2030 suggests numerous opposing factors may be crucial to achieving a sustainable future in an operational reality with many competing forces. The findings also suggest stabilizing QM for rapidly changing settings may be accomplished by nurturing and maintaining positive core aspects.

Aziz, Hussain [24] undertook a qualitative study to compare the obstacles to high-quality education in Pakistan's public and private schools. They selected a sample of 140 teachers (71 from public schools and sixty-nine from private schools) for data collection. To compare the study's variables, the researcher employed a *t*-test. Moreover, according to the survey, Pakistan has difficulty modifying education to meet changing societal needs. Implementing quality management in education throughout the public and private sectors is crucial for the modern day. Compared to primary and higher education, secondary school education is typically overlooked. Youth have been shown to lose the pace at which they picked up knowledge in primary school, providing weaker candidates for entry into universities and colleges. The educational policy concentrates on systemic issues and flaws by determining their causes [66].

Above studies demonstrate the applicability of ISO 9000 and other QM models and programs in educational institutions in several contexts. However, there are issues with its implementation. Few studies have brought to light the appalling conditions that exist in the educational field. This constrained research has highlighted several issues that must be resolved in order to raise educational standards. The issues that came to light throughout these investigations were poor management standards and a lack of governance. A quality management system must be implemented to employ resources effectively and efficiently and boost performance through appropriate checks and balances. Since, there's no previous study available in the literature conducted using QM model ISO 9000 in the same context thus this is the research gap. Accordingly, we planned to conduct a study aims to explore the school principals' perception about quality management practices and its relationship with school culture and student performance in the public secondary schools of Punjab province, Pakistan using QM model, ISO 9000.

## 3 Research methods

**Fig. 2:**
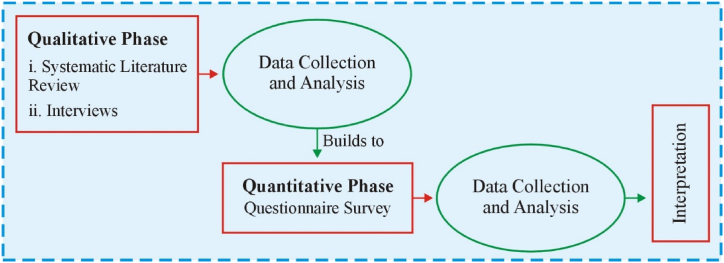
Model showing exploratory mix methods study.

In order to achieve the objectives of the study, the study adopted an exploratory sequential mix-methods research design (Fig. 2). First, this study focused on a systematic literature review (SLR) of the existing studies (63) and interviews with eleven school principals to get their views on quality management and the relationship among three variables: quality management, school culture, and student performance. As school principals currently or had once been responsible for the school's overall quality management programs, they were selected to ensure that they could provide detailed information regarding their experiences and involvement in implementing quality programs. Therefore, the responses were recorded word-for-word based on a semi-structured interview. Next, the data were analyzed via the coding and interpretive assay. The second phase utilized the questionnaire survey and SEM. Initially, SLR findings and qualitative outcomes were employed to design a questionnaire directed toward a large sample, which resulted in the expansion of quantitative construct measures and a theoretical paradigm. The hypotheses in the present study were designed based on the outcomes of the qualitative results (SLR and interviews), which assessed the perception of school principals on quality management practices, school culture, and student performance. Besides, this study also examined the extent to which the quantitative/confirmatory study supported the paradigm of structural relations, quality management practices, school culture, and student performance.

### 3.1 Qualitative phase

At this phase, an SLR of the previous studies was adopted to get the data about the relationship among three variables: quality management practices, school culture, and student performance. An interview guide was prepared according to the dimensions of the ISO 9000 Framework to interview eleven school principals of Punjab province. Thematic analysis phases are shown in Table 1.

**Table 1:** Phases of thematic analysis.

Sr.#	Phase	Description of the Process
1.	Transcribing & Familiarizing with the data	Reading and rereading the data, marking initial thoughts, and transcribing the data
2.	Generating initial codes	Systematically note noteworthy aspects of the data across the full data collection process and compile information pertinent to each code.
3.	Searching for themes	putting codes together into themes, and collecting all information pertinent to each topic
4.	Reviewing themes	Combining codes into themes and gathering all material relevant to each subject
5.	Defining and naming themes	ongoing investigation to improve the details of each theme and the overall narrative that the analysis provides, resulting in distinct definitions and labels for each theme
6.	Producing the report	The last chance for analysis. A scholarly report of the analysis is produced after the final analysis of the chosen extracts, which is then related to the research question and the literature.

Clarke and Braun [67].

### 3.2 Quantitative phase

Several methods exist to solve the research questions and problems, but the researcher should choose the best one that suits his research. So, selecting empirical studies is recommended to get opinions from practitioners working in real-world situations, especially for a large population [68]. This phase involves selecting the targeted population and respondents, designing the sample, developing the instrument, and performing data analysis. A 5-pointed Likert scale Questionnaire was made.

• **Content validity**

In order to examine the transparency and directions of the questionnaire, a team of experts was developed for evaluation purposes. The ambiguity and puzzling statements were excluded or polished to construct the questionnaire properly. Repetition was deleted, and the same ideas were integrated. The content validity of the survey instrument is significant in assessing the content by evaluating the substantial variables [69]. In the present research work, the content analysis was carried out by three experts in the education field (one postdoc researcher at Shanxi Normal University, Xi'an province, China, and two researchers from the University of Agriculture, Faisalabad, Pakistan).

• **Questionnaire readability**

The same experts were asked to check the wording, the clarity of elements, and the variables discussed in the questionnaire. After assessment of the experts, all the modifications are made to the questionnaire as per their comments.

• **Pilot testing of the instrument**

Pilot testing was done to identify the discrepancies in the questionnaire. Ten school principals were involved in this pilot testing process. Firstly, the instrument was received in the light of the view of the experts. Secondly, the instrument was administered to the sampled members (principals of public secondary schools in Punjab province) of the study, and further improvements or changes were made according to the observations of the principals. The pilot study was excluded from the final sample. The results of the pilot studies assist in advancing the survey instrument quality [39,70,71]. The researcher has modified the survey instrument based on the suggestions and comments reported during the pilot study. Based on the pilot study feedback, the questionnaire has been amended and improved for appropriateness and clarity.

• **Administration of the Instrument**

The questionnaire guide was written in both English and Urdu for ease of administration. Urdu might be more understandable because it is the individual's mother tongue rather than English [40,45]. The survey was, therefore, translated into both languages. The language utilized is based on the respondents' preferences to ensure effective and efficient responses. The data collection process took the researcher around five months.

## 4 Data analysis

Partial Least Square-Structural Equation Modelling (PLS-SEM) was used to analyze the quantitative data. For this purpose, the following steps were conducted:

### 4.1 Measurement model for hypothesis

**Fig. 3:**
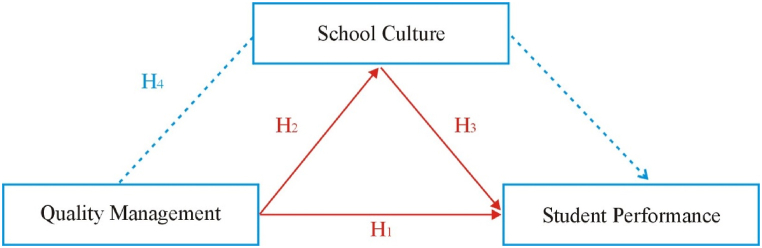
Hypothesis measurement model.

The research model extracted from Phase 1 is presented here in Fig. 3. This model consists of indicators, quality management, school culture, and student performance, which will be used to test the hypothesis.

### 4.2 Assessment of measurement model

Examining the measurement paradigm comprises convergent validity, internal consistency reliability, and discriminant validity [72]. Hair, Black [73] defined internal consistency reliability as the extent to which objects assess the dormant constructs. However, composite reliability (CR) computes internal consistency better than Cronbach's alpha [72]. Although the CR value of '0.6 is acceptable in exploratory research, values above 0.95 indicate redundancy' [74]. The CR scores for all the constructs-Quality Management (0.887), School Culture (0.708), and Student Performance (0.828) in the present study ranged between 0.708 and 0.887 (Table 2), which indicated the presence of the instrument's ICR [75].

**Table 2:** The outcomes of the ICR and CV of the Measurement Model.

The construct of the model	Items	Factor Loading	CR	(Avg.)
Quality Management	QM1	0.803	0.887	0.789
	QM2	0.805		
	QM3	0.821		
	QM4	0.846		
	QM5	0.792		
	QM6	0.684		
	QM7	0.701		
	QM8	0.673		
	QM9	0.669		
	QM10	0.762		
	QM11	0.810		
	QM12	0.772		
School culture	SC1	0.794	0.708	0.504
	SC2	0.876		
	SC3	0.738		
	SC4	0.737		
	SC5	0.703		
	SC6	0.852		
	SC7	0.716		
	SC8	0.818		
	SC9	0.800		
	SC10	0.742		
	SC11	0.751		
Student performance	SP1	0.759	0.828	0.690
	SP2	0.653		
	SP3	0.625		
	SP4	0.719		
	SP5	0.785		
	SP6	0.733		
	SP7	0.678		
	SP8	0.725		
	SP9	0.746		
	SP10	0.781		
	SP11	0.821		
	SP12	0.745		

Hair, Hult [72] defined CV as the 'extent to which a measure correlates positively with alternative measures of the same construct' (p.112). Therefore, the CV could be ascertained based on examining the exterior loading of the pointers' differences and the obtained average variance extracted (AVE) [75,76]. The presence of an increased exterior loading depicted a highly illustrative indicator of the construct. The rule generally states that at least 50 % of each signifier's modification should be elucidated based on dormant variances. Subsequently, an indicator's exterior loading should surpass 0.078 because when squared (0.708), the digit should sum up to 0.50. Nonetheless, overall, 0.7 is viewed as a suitable value for practical reasons [77]. Table 2 shows that the CV is confirmed when the outer loading of all constructs-Quality Management (0.789), School Culture (0.504), and Student Performance (0.690) is adequate and the AVE of all constructs are equalized or have surpassed 0.50 [73].

Furthermore, according to Saleem, Aslam [78], traditional fit indices are statistical tools used to evaluate how well a structural equation model (SEM) aligns with the observed data. One of the most commonly used indices is the Chi-Square Test (χ^2^), which measures the discrepancy between the observed and expected covariance matrices. A non-significant Chi-Square value indicates a good model fit, but this test is highly sensitive to sample size, often leading to significant results in large samples even if the model fits well. Table 3 shows the Chi-square values ≤ 5. Another critical index is the Root Mean Square Error of Approximation (RMSEA), which adjusts for model complexity and aims to measure the lack of fit per degree of freedom. RMSEA values ≤ 0.05 indicate a close fit, values up to 0.08 suggest a reasonable fit, and values above 0.10 indicate poor fit. Table 3 shows the values > 0.5 and < 0.10. The RMSEA also provides a confidence interval, offering a range within which the true RMSEA value is likely to fall, adding further insight into the model's adequacy.

**Table 3:** Goodness of fit statistics for model.

The construct of the model	Items	χ^2^	CFI	TLI	RMSEA	*p-*value
Quality Management	QM1	2.01	0.93	0.91	0.06	<0.01
	QM2	1.98	0.90	0.92	0.07	<0.01
	QM3	1.89	0.90	0.93	0.07	<0.01
	QM4	2.00	0.91	0.91	0.06	<0.01
	QM5	1.87	0.92	0.90	0.05	<0.01
	QM6	1.78	0.93	0.92	0.07	<0.01
	QM7	1.85	0.94	0.93	0.07	<0.01
	QM8	2.01	0.94	0.92	0.06	<0.01
	QM9	1.99	0.91	0.90	0.05	<0.01
	QM10	1.86	0.91	0.90	0.05	<0.01
	QM11	1.86	0.91	0.90	0.05	<0.01
	QM12	1.87	0.93	0.93	0.07	<0.01
School culture	SC1	1.90	0.94	0.91	0.06	<0.01
	SC2	1.98	0.94	0.91	0.06	<0.01
	SC3	1.95	0.93	0.93	0.07	<0.01
	SC4	2.00	0.92	0.90	0.05	<0.01
	SC5	2.01	0.92	0.91	0.06	<0.01
	SC6	1.98	0.90	0.92	0.07	<0.01
	SC7	1.79	0.91	0.90	0.05	<0.01
	SC8	1.79	0.93	0.91	0.06	<0.01
	SC9	1.90	0.94	0.92	0.07	<0.01
	SC10	1.89	0.93	0.91	0.06	<0.01
	SC11	2.01	0.94	0.90	0.05	<0.01
Student performance	SP1	2.00	0.91	0.90	0.05	<0.01
	SP2	1.98	0.90	0.92	0.06	<0.01
	SP3	1.79	0.92	0.93	0.07	<0.01
	SP4	1.87	0.93	0.90	0.05	<0.01
	SP5	1.99	0.93	0.92	0.07	<0.01
	SP6	1.89	0.94	0.92	0.07	<0.01
	SP7	1.90	0.93	0.91	0.06	<0.01
	SP8	2.00	0.92	0.91	0.06	<0.01
	SP9	1.87	0.90	0.91	0.06	<0.01
	SP10	1.79	0.91	0.90	0.05	<0.01
	SP11	1.79	0.93	0.89	0.05	<0.01
	SP12	1.85	0.94	0.90	0.05	<0.01

In addition to these, the Comparative Fit Index (CFI) and the Tucker-Lewis Index (TLI), also known as the Non-Normed Fit Index (NNFI), are widely utilized. The CFI compares the fit of the specified model to a null model (one assuming no relationships among the variables) and values closer to 0.95 or above generally indicate a good fit. Table 3 shows values 0.95 or above for all the items. The TLI, similar to the CFI, adjusts for model complexity and penalizes for overfitting, with values above 0.90 considered indicative of an acceptable fit. Table 3 shows all items having 0.90 and above. The Chi-Square Test (χ^2^) produces a *p*-value that helps determine the model's goodness-of-fit. A high *p*-value (typically above 0.05) suggests that there is no significant difference between the observed data and the model's predictions, implying that the model fits the data well. Conversely, a low *p*-value (typically <0.05) indicates a significant difference, suggesting that the model does not fit the data well. Collectively, these indices provide a comprehensive assessment of model fit, guiding researchers in refining their models to better represent the underlying data structure.

The present study examined discriminant validity (DV) via the Heterotrait-Monotrait ratio of correlations (HTMT). Henseler, Ringle [79] recommended the use of HTMT to evaluate DVs. Based on the predicted factor of correlation, the HTMPT should be < 1.00 (preferably <0.850) to differentiate between the two aspects [80]. The outcomes of HTMT in the present study depicted that the constructs were between 0.440 and 0.816. These digits were dramatically below the point of 1.00 and were established at HTMT. Therefore, Table 4 proved that all constructs were directly interdependent, which fulfilled the discriminant validity selection standards.

**Table 4:** Heterotrait-Monotrait ratio of correlations Criterion.

Construct	Quality Management	School culture	Student Performance
Quality Management	0.751		
School culture	CI.92	0.643	
	(0.502,0.783)		
	0.616		
Student Performance	CI.90	CI.90	
	(0.383,0.792)	(0.477,0.736)	

Besides, the HTMT concluded outcomes also pointed out that the confidence interval (CI) does not show a value of 1 in any of the constructs [79], which also confirms DV. The outcomes of the whole measurement model determined adequate Convergent Validity, Internal Consistency Reliability, and Discriminant validity.

## 5 Results

Results proved that the four hypotheses in the present study were confirmed based on the following data: quality management (H1: β = 0.906, t = 9.850, LL: 0.739, UL: 0.902), which positively connected with student performance. Quality management (H2: β = 0.858, t = 20.127, LL: 0.782, UL: 0.944) was imperatively related to school culture. Besides, the outcomes for the coefficients of PLS depicted that school culture (H3: β = 0.896, t = 8.574, LL: 0.676, UL: 0.799) had a positive impact on student performance. Subsequently, Preacher and Hayes (2004, 2008) suggested an indirect method, which was applied to determine the mediating effect of school culture between quality management and student performance (H4). The analysis portrayed the implicit impact, β = 0.426, which was imperative with a *t-*value of 2.675. The secondary impact of the 95 % Corrected Boot CI Bias: [LL = 0.079, UL = 0.277] did not overlap at 0 and indicated the presence of a mediation [81]. Therefore, the influence of mediation was statistically significant, and school culture (H4: β = 0.426, t = 2.675, LL: 0.079, UL: 0.277) facilitated the connection between quality management and student performance. Hence, Table 5 proved that the results supported H1, H2, H3, and H4, respectively.

**Table 5:** Results of the structural model.

Hypotheses	β	SE	t value	Confidence Level		Decision
				LL	UL	
H_1_: Quality Management → Student Performance	0.906	0.092	9.850	0.739	0.902	Supported
H_2_: Quality Management → School Culture	0.858	0.039	20.127	0.782	0.944	Supported
H_3_: School Culture → Student Performance	0.896	0.078	8.574	0.676	0.799	Supported
H_4_: Quality Management → School Culture → Student Performance	0.426	0.069	2.675	0.079	0.277	Supported

## 6 Discussion

Many empirical studies worldwide have analyzed the implementation of QM practices in K-12 schools. Other research has also examined the impact of overall/total quality management in schools on student academic achievement/success, teacher/student satisfaction and/or motivation, and school effectiveness [82]. Few have examined explicitly the impact of QM practices and relationship to organizational performance, i.e., the student performance, and school culture in a single investigation.

This current research paper's goal is to investigate the growing interest in QM in education and its effect on student performance with mediating effect of school culture in the context of Pakistan's secondary education. The Punjab Government of Pakistan is making wide-ranging efforts to enhance educational quality by taking advanced strides and investing in many programs, including QM initiatives. Despite free education in public schools, student dropouts are rising everyday due to the lower quality of public schools, and the popularity of private schools is increasing in the province. Punjab Schools Reform Roadmap (PSRR) was one of the efforts to resolve these issues in the province. QM serves as one of the three dimensions of this PSRR program. This initiative was launched to improve school quality and enhance student performance in the province. Regarding the evaluation of the consequences and sustainability of the educational reform ten years after its initiation, the outcomes are not adequate.

The study's motivation comes from the Punjab province's secondary schools' implementation of PSRR program and attempt to address the current challenges in terms of educational quality and equity, and the local education system's attention/pursuit to Sustainable Development Goal No. 4 (SDG 4–2030).

Shewhart and Deming's Model for Quality Management in education (1986), Dr. Joseph M. Juran's Quality theory (1904), and the International Organization for Standardization (ISO) 9001 Quality Management System serve as the theoretical foundation for this investigation. The theories intend to increase organizational performance by encouraging employee commitment, teamwork and collaboration, and training and continuous improvement, and highlighting the significance of employee-customer satisfaction throughout the organization. Generally, the QM models serve as the lens through which this study is viewed, as they necessitate an organizational culture of a shared vision and an integrated effort by all organizational members, an emphasis on customers, and effective management-leadership practices in order to ensure quality improvements that, in turn, improve students' academic achievement.

The results of data analysis using structural equation modelling are salient in the confirmation of all four set forth hypotheses. According to the hypothesis summary, significant relationships exist between (1) quality management and student performance, (2) quality management and school culture, and (3) school culture and student performance. Further, the study also found that school culture acted as a complete mediator between quality management and student performance.

A review of extensive literature supports that student performance is predicted by QM practices. For instance, recent studies by literature [46,47] in Nigeria [48], in Bangladesh [49], in Indonesia [50], in Kenya, and [51] in the US revealed that there was a positive relationship between the implementation of QM practices and student academic performance/achievement in the context of public K-12 schools. Further, the impact of quality management of student performance ranges from moderate to strong. The findings of this study are also consistent with those obtained in the related literature [57,60,83,84], which acknowledged a link between school culture and student performance. Prior studies by Glušac, Tasic [57] regarding primary schools in the Republic of Serbia; Pervez, Dahar [61] at secondary level in District Rawalpindi, Pakistan, or Melesse and Molla [60] regarding the case of secondary and preparatory schools in Ethiopia are consistent in their findings that school culture has a significant and positive impact on student academic achievement/performance. Several researchers [[85], [86], [87], [88]] also found that attributes/patterns of school/organizational culture are associated with core QM practices.

Accordingly, the study findings demonstrated that QM features/principles such as effective leadership, teacher engagement in decision-making, quality planning, staff training, and departmental collaboration exert a significant impact on students' academic performance. The studies also argued that QM serves a revolutionary strategy/approach that works well for periodic improvements in student performance, student and staff satisfaction, school competitive advantage, and school sustainability. The approach to QM enhances students' academic progress in a variety of ways, including improving the teaching and learning process, boosting continuous training for education administrators and teachers, stressing school members' satisfaction, fostering collaboration among staff and students, and increasing access to necessary resources.

Similarly, the role of school leadership is emphasized in the employment of QM that establishes a school culture that conveys a clear institutional mission and vision, bolsters quality performance, defines standards for quality, and allocates educational resources for quality performance, all of which enhance teachers' job satisfaction and, as a result, students' academic performance. These findings indicate school management must be aware/cognizant of the actual existence of shared beliefs, values, and customs in their institutions, especially those that are more aligned with QM, for the collective purpose of shifting the organizational culture toward a quality culture.

## 7 Conclusion and implications

The present study surveyed 120 school principals to get their perception about QM practices, school culture and student performance. Besides, this study also examined the extent to which the quantitative/confirmatory study supported the paradigm of structural relations, QM practices, school culture and student performance. This study proved that QM practices improved the school culture and student performance and ascertained that QM practices and student performance showed a positive and significant relationship. Additionally, QM and school culture depict a significant and positive relationship; and school culture and student performance indicate a significant and positive relationship. Finally, the study substantiated that school culture has a positive mediating effect on the relationship between QM and student performance.

The study's preliminary conclusion demonstrated a positive connection between QM and student performance in public secondary schools, and QM was also a significant predictor of school culture. Therefore, efforts taken by the corporate world to the implication of QM would eventually improve school culture and increase the weight of the associated benefits because school culture impacts the overall performance of teachers and students. The results indicated that school culture and QM established a mutually beneficial relationship since quality cannot be successfully administered without considering the school's whole culture. Additionally, this study revealed that school culture directly affects student performance. Also, good school culture is a distinguishing aspect that can bring unmeasured success to an organization. Furthermore, the results also confirmed the role of school culture as the mediator between QM and student performance. This proved that school culture is essential for conveying QM outcomes on student performance in public secondary schools.

The study findings have practical and theoretical implications and recommendations. Educational constituencies might benefit from the results of this study, including educational policymakers, top management of the education department, and school principals. In the same vein with prior studies determining the link between QM, school culture, and student performance, this study claimed effective QM practices would generate positive results/outcomes if secondary schools in Punjab province take advantage of its application for school performance improvement. The present study's findings have significant practical implications at a time when Punjab's educational system is attempting to address the challenges facing local secondary schools in terms of quality and equity that promote competition between public and private schools rather than collaboration.

In that discourse, QM acts as one of the school reform initiatives introduced by the Punjab government to create a revolutionary impact on overall quality management and the teaching-learning process that would contribute to improved school performance in provision of quality education, particularly student performance. Along with this, there should be a focus on improving equity and quality, accelerating institutional reforms, reducing dropout rates, addressing absenteeism, professional development for school administrators and educators, and consolidating public school infrastructure. This act aims to regain community trust in the local education system in terms of educational quality and equity. Moreover, the study provides actionable guidance for improving educational practices within the Punjab province of Pakistan by highlighting the importance of Quality Management (QM) and School Culture (SC). Based on an examination of school principal perceptions in a unique context, the findings of this study contribute to the existing body of knowledge. Further, these findings provide a foundation for scholars and researchers to examine the status of QM in secondary schools throughout Pakistan. This is possible by applying similar frameworks to other regions or educational settings.

The study focuses solely on public secondary schools in Punjab province, Pakistan. While this narrow focus allows for in-depth exploration, it limits the generalizability of the findings to other contexts.

This study is original and essential as there is no previous study dealing with QM issues in the School Education Department by finding the relationship of QM practices, school culture, and student performance in the context of Punjab province, Pakistan.

We propose targeted recommendations designed to enhance practical guidance for educational practitioners considering the insights provided by this study. A wide range of professional development programs should be developed to equip secondary school principals with the necessary skills to lead QM initiatives effectively, and the budget should be allocated more strategically to QM-related projects. Policies must be rigorously implemented and monitored to ensure adherence to timelines and foster an accountability culture. In addition, creating an environment that promotes collaboration and trust among staff members and cultivating a supportive school culture aligned with QM goals are essential. Furthermore, QM policies and planning made for the public schools regarding QM should be tailored regarding the current challenges they are confronting and completed according to time. The top management should be cognizant of change management, i.e., whether new plans should not be imposed until the previous plans get completed since day-by-day changes could confuse the school management with doing its work appropriately. A final step towards maintaining QM practices' relevance and effectiveness is establishing mechanisms for continuous evaluation and improvement. Education institutions can cultivate a culture of excellence and drive positive outcomes by prioritizing these recommendations.

The study findings can be extended in the future by developing an extended model regarding the outcomes investigated in this study. This model can assist the principals and education officers in facilitating their schools with self-evaluation to know the level of quality management in their schools and improve their managerial activities.

## Ethical consideration

The researcher initially secured approval from the Punjab province School Education Department for each school participating in the study. The participants were also given a thorough explanation of the interview and questionnaire. Nobody insisted on taking part in the study or completing the questionnaire. The researcher politely solicited their feedback before disseminating the interview guide and questionnaire.

## Data availability

All the relevant data are included in the manuscript and the supplementary document. No separate repository is attached.

## CRediT authorship contribution statement

**Khalida Parveen:** Writing – review & editing, Writing – original draft, Methodology, Conceptualization. **Tran Quang Bao Phuc:** Writing – review & editing, Validation, Formal analysis. **Abdulelah A. Alghamdi:** Writing – review & editing, Validation. **Tribhuwan Kumar:** Writing – review & editing, Validation, Software. **Sarfraz Aslam:** Writing – review & editing, Visualization, Formal analysis. **Muhammad Shafiq:** Writing – review & editing, Software. **Atif Saleem:** Writing – review & editing, Investigation, Formal analysis.

## Declaration of competing interest

The authors declare that they have no known competing financial interests or personal relationships that could have appeared to influence the work reported in this paper.
