# Supplementary material for: The contribution of quality management practices to student performance: Mediated by school culture
Source: Heliyon. 2024 Jul 19;10(15):e34892. doi: 10.1016/j.heliyon.2024.e34892 (PMC11320296; doi:10.1016/j.heliyon.2024.e34892)
Supplement: Multimedia component 1 [file mmc1.pdf]

Title: The Contribution of Quality Management Practices to Student Performance: Mediated by School Culture

This is a questionnaire prepared for a manuscript to be published in an International Journal. The research study title is “The Contribution of Quality Management Practices to Student Performance: Mediated by School Culture. You have been selected to participate in the study. You are, therefore, kindly requested to answer the following questions below, displaying utmost good faith. The study is purely for academic purposes, and all the information provided will be treated as confidential. Your prompt response will be highly appreciated.

SECTION A: Demographic information

Name of the school: ______________________________
Designation: ______________________________

Gender: Male ☐ Female ☐

Age: 25-35 ☐ 36-45 ☐ 46-55 ☐ Above 55 ☐

Qualification: Masters ☐ Above ☐

Work Experience (years): 0-5 ☐ 6-12 ☐ 13-20 ☐ 21-30 ☐

Category of school: Rural ☐ Urban ☐

Please express your opinion by ticking (☐) the most appropriate box against each statement.

1=strongly disagree, 2= disagree, 3=neutral, 4=agree, 5=strongly agree

**SECTION B: PERCEPTION OF PRINCIPALS OF QUALITY MANAGEMENT PRACTICES**		**1**	**2**	**3**	**4**	**5**
**a.**	**Quality Management**					
QM1	QM improves Employees commitment					
QM2	QM provides Teamwork					
QM3	QM guides actions and processes					
QM4	QM provides a Strategic and systematic approach					
QM5	Quality management provides an integrated system					
QM6	Quality management supports fact-based decisions					
QM7	Quality management supports continuous improvement					
QM8	Quality management provides process thinking					
QM9	Quality management promotes schools’ pride and passion					
QM10	Quality management is viewed as an integral part of planning processes					
QM11	Quality management gives authentic Information & Analysis					
QM12	Quality management supports Human Resource Management					
**b.**	**School culture**					
SC1	The quality Management process is effectively and consistently managed in this school					
SC2	Classroom-based assessment is improved					
SC3	Effective and constant communication is rendered in the school					
SC4	Judgment of teacher competence and performance is done in the school					
SC5	Concentration on teaching processes is enhanced					
SC6	There is a special concentration on the school environment					
SC7	School leaders' strengths are examined appropriately					
SC8	School leaders' weaknesses are evaluated					

SC9	Innovation is found in reporting the results					
SC10	There is the monitoring of quality and equity among students					
SC11	Extracurricular activities are provided					
**c.**	**Student performance**					
SP1	Students are contented with classroom activities					
SP2	Students feel safe on school premises					
SP3	It is easy to approach the facilities at libraries and laboratories for students					
SP4	An innovative teaching-learning environment is designed and implemented					
SP5	Students perceive the teaching methods positively					
SP6	Counseling and supportive learning centers are provided					
SP7	There is an availability of ICT materials and resources in the laboratories					
SP8	Effective instructional strategies and tactics are used in the classroom					
SP9	A good curriculum framework is maintained					
SP10	Effective assessment strategies are available					
SP11	Educational standards are sustained					
SP12	Aspirations for knowledge are provided to the students					
SP13	Creation of personal goals and self-confidence					
SP14	Promoting motivation and study habits					

END OF THE QUESTIONNAIRE

Thank you for taking the time to complete this questionnaire
